# Reappraisal learning curve of laparoscopic Roux-en Y gastric bypass: retrospective results of one hundred and eight cases from a low-volume unit

**DOI:** 10.1186/s12893-021-01058-w

**Published:** 2021-02-15

**Authors:** Hung-Chieh Lo, Sheng-Mao Wu

**Affiliations:** 1 Division of Trauma and Emergency Surgery, Department of Surgery, Wan Fang Hospital, Taipei Medical University, Taipei, Taiwan; 2 Department of Surgery, School of Medicine, College of Medicine, Taipei Medical University, No. 111, Sec. 3, Xinglong Rd., Wenshan Dist., Taipei City, 116 Taiwan, ROC

**Keywords:** Obesity, Roux-en Y gastric bypass, Bariatric, Learning curve

## Abstract

**Background:**

This study aimed to reevaluate the learning curve of laparoscopic Roux-en Y gastric bypass (LRYGB) in the modern era while considering a single surgeon’s experience.

**Methods:**

From the beginning of our LRYGB practice, all patients who met the regional criteria and underwent primary LRYGB were retrospectively enrolled. Patients with a body mass index (BMI) > 50 kg/m^2^ were excluded. Those who underwent surgery in 2016–17, 2018 and 2019 by a single surgeon with 10 + years of laparoscopic experience were assigned to groups A, B and C, respectively. The patient demographics and 30-day outcome data, including the operation time, length of stay (LOS), emergency room visits, readmission, and reoperation, were compared among the groups.

**Results:**

One hundred and eight patients met the inclusion criteria; 36, 38, and 34 patients were assigned to groups A, B and C, respectively. There were no differences in age, sex distribution or common comorbidities among the groups; however, B had a lower BMI (35.1 kg/m^2^ vs. 37.0 kg/m^2^) and a higher rate of hypertension (44.7% vs. 22.2%) than group A. The operation time was markedly reduced (96.1 min and 114.9 min, p < 0.001), and the LOS was shortened (2.2 days and 2.9 days, p < 0.001) in group B compared to group A and remained stationary in group C, with no further reduction in 30-day complications.

**Conclusion:**

The learning process of LRYGB can be shortened to approximately 30 cases if conducted selectively by experienced laparoscopic surgeons. Further follow-up is required to verify the long-term safety and applicability in other patient subgroups.

## Background

The prevalence of obesity has nearly tripled over the past four decades [1]. Bariatric surgery has been shown to be the only treatment with long-term effectiveness for morbid obesity and obesity-related comorbidities [2]. Until recently, laparoscopic Roux-en Y gastric bypass (LRYGB) was acknowledged as the standard bariatric-metabolic procedure and was performed secondarily only after laparoscopic sleeve gastrectomy (LSG) [3]. However, LRYGB is generally considered more technically demanding and has higher complication rates than LSG [4]. Accordingly, a steep learning curve was generally required to meet its safety standards [5]. As the literature often focused on the results of centers of excellence and high-volume centers, only a few studies addressed the learning process in a low-volume center [6].

Traditionally, surgical procedures were mostly learned through observation, on-the-spot assistance or field practice. As time progressed, a new era of surgical education platforms emerged [7]. Online media resources have been proven to be valuable information resources that can improve the learning experience if appropriately used [8] and served as a valuable method in our learning process. Moreover, as learning curves can reportedly be largely reduced based on advanced laparoscopic skills and preemptive bariatric experience [9], the aim of this study was to reappraise the learning curve of LRYGB by a single practicing laparoscopic surgeon with 10+ years of experience in various gastrointestinal surgeries and obtain initial experience performing one-anastomosis gastric bypass (OAGB-MGB) [10]. Meanwhile, for low-volume practices, it is important to maintain safety profiles and carefully audit the results. Our goal was to verify both the peri-operative efficiency in terms of the operation time, length of stay (LOS) and safety regarding all 30-days outcome measures and 1-year weight loss.

## Methods

This retrospective cohort study was conducted at a private university affiliated hospital. From January 2016 to December 2019, all data of consecutive patients who underwent primary LRYGB under the care of a single surgeon were retrospectively collected from a prospectively maintained database after obtaining institutional review board approval (TMU-JIRB No.: N202004071). The patients were eligible for bariatric/metabolic procedures if they met the regional criteria proposed by the International Federation for the Surgery of Obesity and Metabolic Disorders (IFSO) [11]. There are no specific exclusion criteria; however, a body mass index (BMI) > 50 kg/m^2^ was not considered suitable for LRYGB in our initial practice because gastric bypass is generally less effective in these patients [12], and these patients have a higher rate of complications than patients with a lower BMI [13]. In all other patients, the procedure selection among LRYGB, LSG and OAGB-MGB was conducted through a shared decision-making process after full clearance of efficacy and risks based on the literature at the time in the outpatient clinic. The final decision was made using a shared decision-making process after fully considering the potential long-term outcomes of each procedure.

To assess the learning process, the patients were divided into three groups based on their surgery time and case distributions as follows: group A included initial patients who underwent primary LRYGB in 2016–17. Groups B and C included patients who underwent surgery in 2018 and 2019, respectively. All patients underwent complete preoperative evaluations. A simplified and pragmatic enhanced recovery after surgery (ERAS) protocol consistent with the available guidelines at that time that utilized basic and essential changes was fully initiated in January 2017 [14]. In addition to the previous standard of care, bolus dexamethasone (10 mg) and droperidol (0.625 mg) were introduced for postoperative nausea and vomiting prophylaxis. According to the multimodal analgesic regimen, a particular emphasis was placed on the total elimination of opioid medication and its equivalent usage under the aid of laparoscopically guided transversus abdominis plane block. Nasogastric tube, abdominal drainage and urinary catheter placement were discontinued after the ERAS implementation. With routine and on-demand multimodal analgesics and antiemetic agents (i.e., Dynastat, acetaminophen, and ondansetron) postoperatively, discharge was commenced on the first postoperative day if a liquid diet was tolerable.

The recorded parameters, including the patients’ demographic factors and anthropometric data; all relevant outcome measures, including the operation time, LOS and 30-day complications, such as emergency room (ER) visits, readmission, reoperation and conversion; and one year weight loss results were compared between group B and group A and between group C and group B to verify the impact of the learning curve and audit for any quality alterations.

### Surgical technique

LRYGB was performed using a four-abdominal trocar technique and a Nathanson liver retractor. Our technique comprises linear stapling to create a lesser curve-based, 30-ml vertical gastric pouch over a 32 Fr. calibrating tube with 100-cm ante-colic, ante-gastric Roux limb and 100-cm biliopancreatic limb. The enterotomies post both stapling anastomoses were hand-sewn closed. Both mesenteric defects were routinely closed with nonabsorbable sutures. A section of the greater omentum is not routinely performed.

### Data collection and statistical analysis

The Statistical Package for the Social Sciences software version 20.0 (SPSS Inc., Chicago, Illinois, USA) was used to perform the statistical analyses. The descriptive results of the continuous variables are presented as the means ± standard deviations, and the categorical variables are presented as counts and percentages. The categorical data were analyzed using Fisher’s exact test and an unpaired t-test was used to analyze the parametric data when appropriate. The statistical significance tests were two-sided, with a level of significance of 0.05.

## Results

Between January 2016 and December 2019, in total, two hundred and four patients underwent bariatric or metabolic procedures in our unit. Among these patients, three patients underwent a nonprimary procedure, five patients underwent LSG, and 39 patients underwent OAGB-MGB in 2016–17. In 2018, two patients underwent a nonprimary procedure, two patients underwent LSG, and 18 patients underwent OAGB-MGB. In 2019, six patients underwent a nonprimary procedure, one patient underwent LSG, and 20 patients underwent OAGB-MGB. All patients who did not fulfill the inclusion criteria were excluded from the analysis, and ultimately, one hundred and eight patients were enrolled in this study. Of these patients, 36 patients who underwent primary LRYGB in 2016–17 were assigned to group A. The other 38 and 34 patients who underwent primary LRYGB in 2018 and 2019 were assigned to group B and group C, respectively.

The demographic details and clinical characteristics are outlined in Table 1. There were no significant differences between group B and group A with respect to age, sex, preoperative weight or incidence of common comorbidities. However, the patients in group B had a significantly lower BMI (35.1 ± 3.8 kg/m^2^ vs. 37.0 ± 3.6 kg/m^2^; p = 0.03) and a higher rate of hypertension (HTN) (44.7% vs. 22.8%; p = 0.04) than those in group A. Group C did not differ from group B in terms of age, female proportion, or baseline BMI, but group C had a tendency toward a higher rate of diabetes mellitus (32.4% vs. 21.1%; p = 0.28) and a lower rate of HTN (35.3% vs. 44.7%; p = 0.41) than group B. A significantly higher incidence of previous nonbariatric surgeries was found in group C compared to group B (35.3% vs. 23.7%; p = 0.28). However, the main difference was observed among gynecological procedures exclusively for benign lesions. We also did not encounter any severe bowel adhesion required a change in the treatment plan throughout the study.

**Table 1:** Demographic and clinical characteristics of the patients, mean (SD)

	Group A (N = 36)	Group B (N = 38)	Group A vs. B p-value	Group C (N = 34)	Group B vs. C p-value
Age (years)	35.3 ± 10.3	38.5 ± 9.1	0.16	39.6 ± 9.2	0.62
Sex, n (%)					
Male	14 (38.9)	15 (39.5)		15 (44.1)	
Female	22 (61.1)	23 (60.5)	0.96	19 (55.9)	0.69
Preoperative weight (kg)	102.8 ± 16.7	95.8 ± 17.1	0.08	98.7 ± 16.0	0.44
BMI (kg/m^2^)	37.0 ± 3.6	35.1 ± 3.8	0.03*	36.3 ± 3.2	0.13
Comorbidities, n (%)					
Diabetes mellitus	10 (27.8)	8 (21.1)	0.50	11 (32.4)	0.28
Hypertension	8 (22.2)	17 (44.7)	0.04*	12 (35.3)	0.41
Dyslipidemia	18 (50)	21 (55.3)	0.65	18 (52.9)	0.84
Prior operation, n (%)	10 (27.8)	9 (23.7)	0.69	12 (35.3)	0.28
Viceral^a^	3	3		4	
Gyn^b^	9	6		10	

The data are expressed as the means ± standard deviations or numbers and percentages

*BMI* body mass index

^a^All with laparoscopic cholecystectomy and/or laparoscopic appendectomy

^b^Gynecology procedures exclusively for benign lesion

*p < 0.05

The surgical characteristics and outcomes are listed in Table 2. As shown, in the table only one concomitant procedure, i.e., a partial gastrectomy for benign lesions, was carried out in group A. The mean operation time in group B was significantly decreased compared to that in group A (96.1 min vs. 114.9 min, respectively; p < 0.001) and was similar between group B and group C (96.1 min vs. 92.1 min; p = 0.20). The mean LOS was also markedly shortened in group B compared to group A (2.2 ± 0.5 days vs. 2.9 ± 0.8 days; p < 0.001) and was similar, at 2.2 days, between group C and group B (p = 0.70). All procedures were performed with a laparoscopic approach without open conversion. The 30-day complication rate did not differ between groups B and A (2.6% vs. 2.8%; p = 0.97) or between groups C and B (2.9% vs. 2.6%; p = 0.62).

**Table 2:** Surgical perspectives and outcomes, mean (SD)

	Group A (N = 36)	Group B (N = 38)	Group A vs. B p-value	Group C (N = 34)	Group B vs. C p-value
Concomitant procedure, n (%)	1(2.8)	0	0.30	0	
Op time (min)	114.9 ± 29.1	96.1 ± 13.5	< 0.001*	92.1 ± 12.1	0.20
LOS (days)	2.9 ± 0.8	2.2 ± 0.5	< 0.001*	2.2 ± 0.4	0.70
Conversion	0	0		0	
30-day complications, n (%)	1 (2.8)	1 (2.6)	0.97	1 (2.9)	0.62
Stenosis	1	0		0	
Melena	0	0		1	
Hematemesis	0	1		0	
30-day ER visit, n (%)	0	2 (5.3)	0.16	1 (2.9)	0.62
30-day readmission, n (%)	1 (2.8)	1 (2.6)	0.97	0	0.34
30-day mortality, n (%)	0	0		0	
1-year follow-up, n (%)	30(83)%	30(79)%		28(82)%	
%EWL	76.4 ± 18.2	80.6 ± 19.8	0.40	82.4 ± 14.3	0.70
%TWL	26.7 ± 5.1	25.1 ± 6.6	0.28	28.0 ± 5.4	0.07

*Op* operation, *LOS* length of stay, *ER* emergency room, *%EWL* percentage of excess weight loss, *%TWL* percentage of total weight loss

*p < 0.05

In total, five patients experienced 30-day adverse events, and three of these cases were classified as complications. One patient in group A was readmitted for gastrojejunostomy stenosis on postoperative day 30, which was relieved following a single session of balloon dilatation. In group B, two patients visited the ER after discharge as follows: one patient visited for nonspecific focal abdominal pain, and the other patient, who visited for hematemesis on postoperative day 9, was readmitted and recovered uneventfully after proper medical treatment. In group C, one patient was noted to have self-limiting melena that subsided under supportive treatment. Another patient visited the ER for lower back pain. The rates of 30-day ER visits and 30-day readmissions did not differ among the groups. There were no reported cases of anastomotic leakage, reoperation or mortality throughout the study period.

Up to 12 months postoperatively, 83% of the patients in group A, 79% of the patients in group B and 82% of the patients in group C were available for follow-up. No statistically significant differences in the percentage of total weight loss (%TWL) and percentage of excess weight loss (%EWL) were found. The %EWL was 76.4%, 80.6%, and 82.4% and the %TWL was 26.7%, 25.1% and 28.0% in groups A, B and C, respectively.

## Discussion

Here, we report the outcomes of 108 initial patients who underwent LRYGB over a 4-year period in a low-volume hospital, indicating the learning curve. By comparing the results among the 3 groups, significant improvements in the operation time and LOS with an acceptably low rate of complications were observed after the initial 36 cases (group A). The present study demonstrated that the learning curve of LRYGB can be safely reduced to 30 + cases in the modern era under a unique setting.

Because of increasingly complex techniques and dependence on advanced instruments, the acquisition of new laparoscopic skills is considered difficult. When conducting LRYGB in morbidly obese patients, several other inherent technical barriers, such as body habitus, multistep reconstructive procedures involving multiabdominal quadrants and laparoscopic suturing and knot tying skills, are likely to occur. Therefore, this procedure was once rated as a 9.5 on a difficulty scale of 10, indicating substantial technical difficulty [15]. These skill-related prerequisites can result in adverse consequences during the early phase of practice, especially in a low-volume practice [6]. Traditionally, various educational programs, such as workshops [16], bariatric fellowships [17] and systematic training programs [18], have been available to facilitate this process. In recent years, new-era platforms have emerged, providing another type of auxiliary training approach [7]. Their popularity among medical professionals has increased, as these platforms generally enable more visual and auditory interactions than journals or textbooks [19]. A systematic review of the impact of e-learning demonstrates significant gains in knowledge compared with traditional teaching patterns [20]. In our self-learning process, in addition to traditional learning methods, these online multimedia materials provide considerable references and guidance despite the lack of objective tools for gauging their impact.

By considering a single surgeon’s perspective, we retrieved comparative data from the literature discussing the relevant process by a single surgeon (Table 3). Among them, variability in surgical techniques exists. Moreover, several modifications of traditional methods have occurred; for instance, the retro-colic placement of the Roux limb [21, 22] or circular-stapled anastomosis [21, 23, 24] have largely fallen out of favor. Meanwhile, there are differences among studies in terms of backgrounds, annual hospital volumes and former laparoscopic/bariatric experience levels. Despite the undisputable importance and heightened awareness of proper fellowship training [25], there is no standard teaching approach, and accredited bariatric programs are not globally available. For example, while carried out during different periods, some hospitals conducted other laparoscopic bariatric procedures [9, 26] or performed open bariatric surgeries [22], while other hospitals just started after complete fellowship training [23, 27] or are based solely on advanced laparoscopic skills [21, 24, 28]. Additionally, the case selection criteria considerably differed. In general, fellowship-trained bariatric surgeons or those conducted the procedures after preceding bariatric experience appear to have a shorter learning curve and implement a more efficient practice. For instance, after completing a month-long mini-fellowship, Shen et al. achieved a considerably decreased complication rate and proficiency after only 30 cases [27]. In particular, these authors utilized the case selection criteria following the IFSO Asian-Pacific guidelines, which is similar to our research [11]. However, these authors reported an initial complication rate of 26.7%, which included 6.7% conversion, 10% reoperation and 5% leakage rate. While surgeons at high-volume hospitals often have the opportunity to master the procedure in a short period with preferable results [29], Shin et al. participated as assistants in 30 surgeries and conducted the first few surgeries under proctoring. These authors analyzed their first one hundred cases within 5 months and concluded that the learning curve plateaued after 50 surgeries [23]. While these authors realize a marked decrease in the operation times (113 min pre- and 73 min post-learning curve), they indicated that there was no further notable reduction in complications after the learning curve. With particularly vast prior experience in LAP BAND ®, Ballesta-Lopez et al. also published a large series with marked decreased operative time and LOS after the first 100 surgeries [26]. Particularly, as one of only two studies, in addition to the study by Andrew et al. [22], which was conducted with totally hand-sewn gastrojejunostomy, the authors reported no negligible leakage rate of up to 9%, a 5.1% reoperation rate (mostly for leakage) and a 29.2% complication rate. In other studies with only prior advanced laparoscopic experience [21, 24, 28], the learning curve was slightly longer, and there was a substantially prolonged operation time compared with studies involving surgeons who completed a fellowship [9, 23, 27]. For instance, Oliak et al. reported a series with the highest mean BMI of 51 kg/m^2^ and proposed that the learning curve plateaued after 75 surgeries [21]. The surgeons’ operative times substantially decreased from 189 min during the first 75 cases and then gradually decreased. Notably, the perioperative complication rates were substantial among all studies both before (32%) and after the learning curve (15%). Nguyen et al. evaluated 150 consecutive cases, with the longest mean operation time of 250 min before the learning curve; these authors also observed that an initial lack of experience (< 75 cases) was a major factor associated with major complications and an increased reoperation rate [24]. However, with advanced laparoscopic skills and preemptive bariatric experience, Agrawal et al. suggest that LRYGB can be performed safely with a minimal complications rate of only 1.4% and effectively without any learning curve required [9]. Notably, our initial results stabilized after only 36 surgeries and we had a low initial 30-day complication rate of 2.8%. The only difference is that their research was conducted after the completion of fellowship training [9]. However, consistent with these aforementioned studies, a sharp improvement in terms of the operation time was observed in the current study after the learning curve. Similar to the findings reported by Shin et al*.* [23]*,* we observed no further decrease in the complication rate after the learning curve. Only one patient presented with stenosis, and two other patients presented with hemorrhagic complications as follows: one with hematemesis and one with melena. Considering the reported stenosis rate of 2.2 to 10% [21, 23, 27, 28] and that the hemorrhagic complication rate ranges from 1 to 3.3% during the learning process [23, 24, 28], we deemed our initial results acceptable. Furthermore, there were no cases of mortality, leakage, conversion or other major complications. While prolonged hospital stay is not uncommon after such a procedure. Nguyen et al. noted that 11% of their cases had an LOS over 4 days [24], and the LOS reportedly ranges between 6 to 6.4 days before the learning curve and 4.8 to 5 days after the learning curve [27, 28]. We observed a notably shorter LOS, with a mean of 2.9 days before the learning curve and 2.2 days after the learning curve. In addition, only 2.8% of our patients required hospitalization for more than 3 days. Our learning curve and timespan to reach competency are consistent with the findings of a systemic review that reported between 30 and 70 surgeries [30]. Notably, the consistent outcome obtained in group B and group C can be considered an early achievement of proficiency because we reached this goal within less than 70 accumulative surgeries, while historically, between 70 and 150 surgeries are usually required [30].

**Table 3:** List of historical studies involving a single surgeon

Authors	Study period	Background	Patients (n) Groups inclusion criteria	Age^a^ (years)/BMI^a^ (kg/m^2^)	Learning curve	Mean op time (min)		Complications (%)	
						Pre-LC	Post-LC	Pre-LC	Post-LC
Agrawal et al. [9]	10–11	Advanced scope fellowship laparoscope bariatric	74 1 group primary procedures	45.1 47.7	Not required	160	n/a	1.4	n/a
Shen et al. [27]	09–11	Advanced scope fellowship	60 2 groups IFSO-APCcriteria^b^	34.2 41.5	30	120	80	26.7	6.7
Shin et al [23]	03	Advanced scope fellowship	100 2 groups unselective	42.6 47.6	50	113	73	32	8
Oliak et al. [21]	99–01	Advanced scope	225 3 groups primary procedures	40 51	75	189	125	32	15
Huang et al. [28]	05–07	Advanced scope	100 2 groups unselective	31.2 43	50	217	105	15	3
Nguyen et al. [24]	n/a	Advanced scope	150 2 groups unselective	40 47	75	250	n/a	12	1
Andrew et al. [22]	02–04	Open bariatric	201 3 groups unselective	37 49.2	70	145	118	19.4	11.9
Ballesta-Lopez et al. [26]	00–04	Advanced scope LAP BAND®	600 6 groups unselective	38.7 44.4	100	166	109	29.2	14
Current study	16–19	Advanced scope laparoscope bariatric	108 3 groups primary procedures	37.8 36.1	30 +	115	96	2.8	2.6

*Op* operation, *BMI* body mass index, *n/a* no data available

^a^Mean

^b^IFSO-APC criteria refer to the International Federation for the Surgery of Obesity and Metabolic Disorders Asia Pacific Chapter criteria[11]

Many factors likely contributed to our early desirable results. First, in contrast to most studies with an unselective patient approach [22–24, 26, 28], patients with a BMI > 50 were preferably offered alternative treatment modalities considering safety and long-term effectiveness [12, 31]. As a result of this selective approach, the mean BMI of 36.1 kg/m^2^ in our series was significantly lower than that in former studies, which ranged from 43 to 51 kg/m^2^ [21, 28]. Second, as guidelines for tailored peri-operative care were established in 2016 [14], a pragmatic enhanced recovery protocol was gradually adopted in our unit. Furthermore, our preceding experience with OAGB-MGB may have transferred to subsequent LRYGB and increased its safety [10]. Similarly, comprehensive care and improved techniques have been demonstrated in other studies across different periods [32]. Because only two aforementioned studies included cases after 2010 [9, 27], we believe that general improvements further contribute to this desirable result.

### Limitations

Limited by the selective approach, our result may not be generalizable to all patient subgroups. Nevertheless, a desirable outcome can be accomplished during the learning process via this selective approach. Moreover, because of the retrospective design and nonrandomized nature, the presence of clinical heterogeneity among the groups may compromise their comparativeness.

## Conclusion

In conclusion, the current study shows that a satisfactory learning curve can be safely accomplished in a low-volume center with the complication rates, operation times and LOS plateauing after 30 + cases. Additional data regarding long-term efficacy, safety and generalizability are required.

## Data Availability

The datasets generated and/or analyzed during the current study are not publicly available due to restrictions by the local Institutional Review Board but are available from the corresponding author upon reasonable request and with permission from the local Institutional Review Board.

## Abbreviations

LRYGB: Laparoscopic Roux-en Y gastric bypass
BMI: Body mass index
LOS: Length of stay
LSG: Laparoscopic sleeve gastrectomy
OAGB-MGB: One-anastomosis gastric bypass
IFSO: International Federation for the Surgery of Obesity and Metabolic Disorders
ER: Emergency room
HTN: Hypertension
